# Trench Fever: A Diagnostic Dumping Ground?

**Published:** 1916-03-11

**Authors:** 


### TRENCH FEVER.

### A Diagnostic Dumping Ground?

For a long time there has been a disposition among the officers of the Royal Army Medical Corps in France and Flanders to adopt the conveniently vague label "trench fever" as a diagnosis for large numbers of cases of brief and non-serious pyrexial illness which could not be clearly and speedily made out to conform to some definite and recognised malady. "Trench fever," in fact, has served in the British Expeditionary Force very much the same office that "influenza" does in civil practice at home: that is, to cover all sorts and conditions of illness for which time and other facilities might not allow of full and accurate classification. In the South African campaign a similar dumping ground for unsorted fevers was popularised under the initials S.C.F., which stood for "simple continued fever." The mystery of simple continued fever was never cleared up, though thousands of cases were thus diagnosed. Probably many of them were mild cases of enteric (typhoid or paratyphoid were then undifferentiated), or of enteric modified by previous inoculation.

### A Comprehensive Category.

In like manner there is no doubt in the minds of observant officers of the Royal Army Medical Corps that the term "trench fever" has been used to include all sorts of diseases. A considerable proportion of these have turned out on searching bacteriological examination to be cases of the paratyphoid fevers; and a few to be true typhoid modified by inoculation. Others have been shown to be rheumatic fever, appendicitis, and other diseases: a good many have certainly been true influenza. But a great many cases have never been rigorously investigated by laboratory methods, and many hundreds, if not thousands, have been diagnosed "trench fever" and allowed to remain in that sweeping category. The result of all this diagnostic confusion is that some medical officers have protested against the name "trench fever" altogether, alleging that no such disease has been proved to exist, and that the name merely serves to cloak ignorance and to hinder full investigation. No doubt very many of those thus labelled can be shown to suffer in reality from some definite disease already familiar to medical science: to be, in fact, misdiagnosed. Some officers say there is also a hitherto undescribed malady, for which the name "trench fever" is appropriate: others are not satisfied about it. Admittedly it is strange that a new disease should spring up so suddenly in a country and climate as familiar as France; and admittedly the onus of proof lies upon those who assert the existence of such a disease, not upon those who are still sceptical.

Practically the first attempt to supply such proof* is contained in an interesting paper lately published by Captain J. W. McNee and Lieut. Arnold Renshaw.† These two observers are quite convinced that "trench fever" does exist as a definite clinical entity; and that it is typically a relapsing fever, of which they describe two clinical types. They are positive that they have succeeded in eliminating the paratyphoids, and that all their cases of "trench fever" have been definitely negative to the usual tests for these diseases (Widal, blood culture, fœcal culture). They find that two categories of men alone suffer from the malady; namely, men from the fire area—trenches, artillery positions, etc.—and men of the R.A.M.C. No cases occur amongst Army Service Corps units, ammunition columns, and troops not actually in or near the trenches. The liability of the R.A.M.C. is naturally attributed to infection from the patients under treatment.

Space does not permit of a full analysis of the cases described by McNee and Renshaw. It must suffice to say that several of the cases of their first type bear a strong family resemblance to paratyphoid, and that no one with wide experience of this disease would care to exclude it from the diagnosis on the strength merely of negative bacteriological findings. Clinically it is not apparent that sufficient care has been taken to exclude the paratyphoids, though quite possibly this impression, gained from the particulars vouchsafed, may be in fact a mistaken one. When we come to the second clinical type, the authors appear to be on much firmer ground. Here a relapsing fever of a pretty definite type, clinically speaking, does really seem to have been encountered. The doubt as to the identity of their two types, occurred to McNee and Renshaw; but they reject the idea as disproved by their experimental work, which was admirably thorough and searching. It is true their findings are mainly negative, but they succeeded in transmitting the disease by injecting blood from sufferers into healthy men who volunteered for the experiment, and they succeeded in changing one type of the disease into the other. As a result of careful experiments they conclude that the virus does not reside in the plasma or serum of the blood, but in the corpuscles (either red or white): it cannot pass a filter, but all efforts to find a parasite have failed. Blood films give no real help in diagnosis, which is really founded bacteriologically on premisses negative to other diseases. They suggest that the disease is either contagious man to man; or, more likely, carried by one or more of the insect plagues of the trenches.

Lastly, it is just worth mentioning Sir Wilmot Herringham's curious misconception that the name "trench fever" was used for the first time last November by Hunt and Rankin. As a matter of fact the phrase has been common at the Front certainly since last May, though we are not in a position to say who invented it, or when it was first made use of.

* Except an unconvincing paper by Hunt and Rankin last November.

† *British Medical Journal*, February 12, p. 225.

